# Screening and Characterization of *Streptomyces* spp. Isolated from Three Moroccan Ecosystems Producing a Potential Inhibitor of the Drug Efflux Pump AcrAB-TolC

**DOI:** 10.3390/biotech11030022

**Published:** 2022-06-29

**Authors:** Asma Azmani, Sanaa Lemriss, Mustapha Barakate, Amal Souiri, Driss Dhiba, Lahcen Hassani, Hanane Hamdali

**Affiliations:** 1 Laboratory of Agro-Industrial and Medical Biotechnologies, Faculty of Sciences and Technology, University of Sultan Moulay Slimane, P.O. Box 523, Beni-Mellal 23000, Morocco; a.azmani@gmail.com (A.A.); slemriss@lram-fgr.ma (S.L.); asouiri@lram-fgr.ma (A.S.); 2 Department of Biosafety PCL3, Laboratory of Research and Medical Analysis of Gendarmerie Royale, Rabat 10090, Morocco; 3 Cadi Ayyad University, Faculty of Sciences Semlalia, Laboratory of Microbial Biotechnologies Agrosciences and Environment (BioMAgE, Labelled Unit CNRST N°4), P.O. Box 2390, Marrakech 40000, Morocco; mbarakate@uca.ma (M.B.); lhassani@uca.ac.ma (L.H.); 4 University Mohammed 6 Polytechnic (UM6P), International Water Research Institute (IWRI), Moulay Rachid, Ben Guerir 43150, Morocco; driss.dhiba@um6p.ma

**Keywords:** efflux pumps inhibitor, molecular identification, screening, *Streptomyces*

## Abstract

Traditional antimicrobial antibiotics are increasingly suffering from the emergence of multidrug resistance among pathogenic microorganisms. The antibiotic era is threatened by the ruthless rise of resistance in bacterial infections. A significant role in these resistance profiles is attributed to multidrug efflux pumps. Hence, much effort is being directed towards developing new compounds to overcome this problem. During our screening program of efflux pumps inhibitors (EPI) produced by bioactive Moroccan Actinobacteria, 210 isolates were screened for their antibacterial activities against *Escherichia coli* strains containing a system of efflux pump AcrAB-TolC, fully functional, and its mutant, inactivated due to the insertion of transposon Tn903 in AcrAB operon, using the method of agar disc diffusion. The results showed that 14 isolates were able to produce EPI as they were active against the wild type strain but not against the mutant in comparison with the synthetic inhibitor L-Phe-L-Arg-β-naphthylamide (PaβN). We focused on the highest EPI activity produced by four strains (Z332, Z35/G, Z385/b and 136). Taxonomic studies and the 16S rDNA sequence indicated that these strains belonged to the *Streptomyces* species. This work could contribute to the discovery of a new class of antibacterial agents that could expand the therapeutic arsenal.

## 1. Introduction

The discovery of antibiotics has been the most important scientific breakthrough of the 20th century. Antibiotics were being used to fight infections, and several bacterial diseases were considered to be on their way to eradication [1]. However, intense antibiotic use has promoted the emergence of antibiotic-resistant pathogens, one of the biggest global public health issues because serious infections become extremely difficult to treat [2].

The basic mechanisms of antibiotic resistance include the inactivation of the antibiotic, alteration of the bacterial target, reduction of antibiotic permeability into the cell, development of a resistant biochemical pathway (sulfonamides), and multidrug efflux pumping [3]. In addition, it could occur as the result of mutation or the acquisition of exogenous resistance genes, or increased efflux, responsible for antibiotic extrusion to the outside [4]. Among these main mechanisms, a significant role is attributed to multidrug efflux pumps [5].

In fact, these systems are classified into five families, can induce specific resistance to a single antibiotic class or to a large number of antibiotics, which confer a multi-drug resistance (MDR) phenotype to bacteria [6]. Hence, there is a resurgence of interest in the identification and development of potent agents that could be able to overcome the existing resistance, or inhibitory substances that may block efflux pumps and restore the antibiotic drugs’ susceptibility.

Despite the supremacy of synthetic antimicrobial drugs, more recently, efforts have refocused on discovering new natural-product antibiotics because several of these synthetic compounds are not applicable to clinical use due to their toxic properties [7]. Until recently, the majority of antimicrobial compounds in current use for the treatment of various infectious diseases were isolated from microorganisms [8]. Among these, Actinobacteria are viewed as a major source of bioactive natural products [9], especially the genus *Streptomyces* [10]. Furthermore, the exploration of new soils and habitats from extreme environments is one of several research programs established to obtain new strains and new bioactive metabolites [11].

Thus, in our study, we focused on the search for bioactive compounds produced by Actinobacteria isolated from three Moroccan ecosystems. Through the screening of isolates producing natural inhibitors of AcrAB-TolC pumps of *Escherichia coli* [12], a tripartite assembly in the bacterial membrane conferred resistance to a broad spectrum of antibiotics. In addition, we performed culture-based, morphological, biochemical and molecular 16S rRNA gene sequence methods to identify the most active strains.

## 2. Materials and Methods

### 2.1. Collection of Actinomycete Strains

The 210 Actinobacteria isolates used in this study were from the collection of the Laboratory of Biology and Biotechnology of Microorganisms, Cadi Ayyad University, Marrakesh, Morocco. They were isolated from various Moroccan habitats including rhizospheric soils and endophytes of endemic aromatic and medicinal plants [13,14]. All strains were maintained in glycerol (20%) at −20 °C.

### 2.2. Collection of the Tested Bacterial Strains

To test the ability of the collection of Actinomycete strains to produce efflux pump inhibitors (EPI), we used four bacteria. The *E. coli* (AG100, AG100A) and *Staphylococcus aureus* (SA 1199 & SA 1199B) test strains used in this study were obtained from the Microbial Strain Collection of UMR-MD1 (membrane transporters, Chimioresistance and Drug-Design, Faculty of Medecine and Pharmacy, Marseille, France) [12]. *E. coli* AG100 is a wild-type strain containing a system of efflux pump AcrAB- 84 TolC that is fully functional [12], and *E. coli* AG100A has the pump system AcrAB- 85 TolC efflux inactivated due to the insertion of transposon Tn903 in AcrAB operon; *S. aureus* sensitive strain SA-1199 and its mutant are resistant to fluoroquinolones, and SA-1199B is also called *S.aureus* NorA [12]. The SA-1199B strain has a mutation in the promoter, 89 bp upstream of the start codon of norA gene, and a mutation Ala116 Glu in the QRDR of GrlA gene (parC) [15].

### 2.3. Determination of the Efflux Pumps Inhibitors from the Selected Strains

For the identification of the efflux pumps inhibitors that could be synthesized by the Actinobacteria isolates, we measured the Minimum Inibitory Concentration (MIC) and Minimum Bactericidal Concentration (MBC) of several antibiotics in order to select the required antibiotics and their concentrations. The MIC is defined as the lowest concentration of the dilutions of an antibiotic to inhibit the growth of the test strains, and the MBC is the lowest concentration of an antibiotic required to kill it. The MIC of antibiotics as well as of Actinobacteria was determined by the microdilution method using a microtiter plate [12]. The antibiotics used were cloxacillin, flucloxacillin, chloramphenicol, thiamphenicol, ciprofloxacin, and norfloxacin. The obvious reduced permeability of antibiotics in some bacteria has been credited to the constitutive expression of efflux pumps, which confers a natural resistance to several antibiotics. Therefore, a wide spectrum or high-level resistance can be detected in bacteria in which active efflux and other mechanisms of resistance function synergistically. This is exemplified in an *E. coli* strain that in parallel expresses β-lactamase and efflux pumps, and is consequently also insensitive to β-lactams resisting enzymatic hydrolysis [16]. All the antibiotics were serially diluted in Muller Hinton broth (Merck KGaA, Darmstadt, Germany), and the inoculum preparation was made by a direct broth suspension of isolated colonies selected from an 18 to 24 h agar plate. Then, in each microwell containing 100 µL of the antibiotic, 100 µL of a bacterial inoculum of 10^5^ CFU/mL was added. The test plates were incubated at 37 °C for 18 h.

### 2.4. Screening of Actinomycetes Producing Efflux Pump Inhibitors (EPI)

The screening of Actinobacteria isolates for their ability to produce EPI was performed by the agar diffusion method [17]. First, pure Actinobacteria isolates were grown on Bennett’s medium (beef extract (Merck) 1 g/L; glucose (Merck) 10 g/L; peptone (Merck) 2 g/L; yeast extract (Merck) 1 g/L and agar (Difco) 15 g/L). After incubation for 7 days at 30 °C, mycelia plugs (9 mm in diameter) were cut and placed on Muller−Hinton agar plates (Merck) [12] supplemented with the antibiotic to be tested at a concentration equal to MIC/2 and which were previously seeded with the appropriate test organism. Plates were first kept in a refrigerator (4 °C) for at least 2 h to allow the diffusion of any produced EPI, then incubated at 30 °C. Inhibition zones were determined after 24 h, and only isolates that showed a diameter of inhibition larger than 10 mm were considered active. All isolates were tested in three independent replicates, and the Phe-Arg-β-naphthylamide (PAβN), a broad-spectrum inhibitor, was used as the positive control. This inhibitor is known to block the efflux systems of many bacteria [18]. Moreover, this EPI alone restores the activity of several families of antibiotics, including fluoroquinolones, macrolides and tetracyclines [19].

Furthermore, in order to confirm the initial screen, antibacterial activities of the isolated strains were performed by using the agar well-diffusion method [20]. Actinobacteria strains were grown for 7 days at 28 °C in the Bennet production medium. Two to three identical colonies were picked from the plate and transferred to the broth. Each strain to be tested was grown overnight in LB media, and its OD600 was determined [20]. Seven mm (diameter) wells were perforated in the agar, and 50 μL of each Actinobacterial culture were poured into the well. Plates were subsequently incubated at 28 °C. Inhibition zones were measured after 24 h of incubation. 

### 2.5. Culture-Based and Morphological Characterizations of the Selected Strains

The cultural features of the strains were characterized following the instructions given by the International *Streptomyces* Project (ISP) 20 media, namely yeast-malt agar (ISP2), oatmeal agar (ISP3), inorganic salt-starch agar (ISP4), glycerol asparagine agar (ISP5), peptone yeast extract iron agar (ISP6) and tyrosine agar (ISP7) [21]. The selected strains were first identified according to traditional morphological criteria, including the characteristics of colonies on the plate, morphology of substrate and aerial hyphae, morphology of spores and produced pigments [22].

### 2.6. Physiological and Biochemical Characterization of the Selected Strains

The physiological testing holds three different approaches for the characterization of Actinobacteria strains. The resistance toward sodium chloride test is taken by analyzing the growth on basal medium with 0, 2.5, 5, 7.5, and 10% of sodium chloride [23]. The utilization of 10 different carbon sources was determined on the basis of the 20 methodology using a microplate technique with twelve well plates. Commercially available test kits such as ApiZym^®^ (bioMérieux, Marcy-l’Étoile, France) were used for the biochemical characteristics of the strains. The Api stripes were inoculated following the manufacturer’s manual directions [23].

### 2.7. Amplification and Sequencing of the 16S rDNA of the Selected Strains

Genomic DNA was isolated from pure cultures in Bennet agar medium at 37 °C for four selected strains (Z332, Z35/6, Z385/6 and 136) and was extracted using the Maxwell^®^ RSC Instrument (Promega, Madison, WI, USA) and the Maxwell^®^ RSC PureFood GMO and Authentication Kit (Promega) according to the manufacturer’s recommended protocol. PCR amplification of the 16S rDNA was performed using two primers: 27f (5′-AGAGTTTGATCCTGGCTCAG-3′) and 1492r (5′-GGTTACCTTGTTACGACTT-3′) [24]. The 16S rDNA was amplified by PCR using AccuPower Taq PCR PreMix (GE Healthcare, Little Chalfont, UK). The amplification was performed on a GeneAmp PCR 9700 System (Applied Biosystems) thermal cycler according to the following protocol: after initial denaturation (96 °C for 1 min), 30 cycles of 96 °C for 30 s, 60 °C for 30 s and 72 °C for 1 min 30 s were performed, followed by a final extension (5 min, 72 °C). PCR products visualized on a 2% (*w*/*v*) agarose gel stained with ethidium bromide were sequenced bidirectionally with 27F and 1492R primers using a Sanger sequencer.

Sequences’ similarities were performed using the online sequence analysis resources LEBIBI database [25] and GenBank through Nucleotide BLAST (http://www.ncbi.nlm.nih.gov/BLAST/, accessed on 8 April 2021). Unrooted phylogenetic trees were inferred using the Neighbor-Joining method [26]. The percentage of replicate trees in which the associated taxa clustered together in the bootstrap test (1000 replicates) is shown next to the branches [27]. The evolutionary distances were computed using the Kimura 2-parameter method [28] and are in the units of the number of base substitutions per site. Evolutionary analyses were conducted in MEGA X [29].

## 3. Results

### 3.1. Primary Screening for Antibiotics Selected for Determination of Efflux Pumps Inhibitory Activity by Actinobacteria

After assessing both MIC and MBC data, the antibiotics cloxacillin and chloramphenicol displayed very different reactions between wild type and mutant strains. Consequently, these two antibiotics were used for the screening procedure. Table 1 summarizes all the results obtained for the MIC and MBC of six antibiotics tested against two test bacteria and their mutants. As a result, we found that for *S. aureus* and *E. coli,* cloxacillin (or flucloxacillin) and chloramphenicol showed, respectively, MIC and MBC that were very different between wild strains and mutants. Thus, our choice was fixed on these two antibiotics for the screening of the Actinobacteria able to produce substances with efflux pumps inhibitory activity.

### 3.2. Screening for Actinobacteria Showing an Efflux Pumps Inhibitory Activity

For our screening, a total of 210 Actinobacteria isolates were analyzed for their ability to produce antimicrobial activities against the test strains. The inhibition diameters were measured (Table 2). Among the 210 isolates screened, 17 displayed efflux pump inhibition and 190 did not.

Thus, 9.52% of isolates could potentially produce EPI substances; 6.67% were active against Gram negative bacteria (AG100) and 2.86% against the Gram-positive bacteria (SA 1199B). According to the data shown in Table 2, only four of the 14 actinobacteria strains exhibited a high efflux pumps inhibitor activity, in particular against Gram negative bacteria in comparison with the Gram positive ones. The reason behind this sensitivity could be attributed to morphological and chemical composition properties like the membrane of Gram positive bacteria having lipopolysaccharides, which confer impermeability to bioactive substances to the cell wall [30].

The obtained results using the confirmatory assay of the agar well diffusion method agree with the results of the test diffusion on the agar disc (Table 3). In fact, the 14 selected Actinobacteria strains showed a high efflux pumps inhibitor activity, specifically against Gram negative bacteria in the presence of chloramphenicol, and with cloxacilin for the Gram positive ones (Table 3).

### 3.3. Characterization of the Active Strains

Among the 17 isolates collected from a Moroccan rhizospheric soil showing an EPI activity, we selected four strains, Z332, Z35/6, Z385/6, and 136, based on their strong activity against the tested strain of *E. coli*. We have done a preliminary identification using phenotypic characterization (Table 4).

The resistance towards sodium chloride is a helpful tool in differentiating between species of Actinobacteria. The four selected strains exhibited a salt tolerance of up to 7.5% with an optimum growth at 5% NaCl; hence, these strains could be placed in the intermediate salt tolerance group.

Actinobacteria form a significant group of microbial populations in soil, able to produce many valuable enzymes that can decompose a variety of organic materials and that could be applied in different industries. The API ZYM test method is a simple system used in order to detect selected enzymes in *Streptomyces* species (Table 4).

According to the results of the strain Z332, a positive reaction was found for several enzymes, such as alkalinephosphatase (level 5), esterase (level 2), esterase-lipase (level 3), lipase (level 2), leucine arylamidase (level 5), valine arylamidase (level 4). Additionally, the strain Z385/b cannot produce alpha and beta galactosidase, alpha glucuronidase, alpha mannosidase, and alpha fructosidase.

Furthermore, the strain 136 is able to produce alkalinephosphatase (level 5), esterase (level 2), esterase-lipase (level 4), lipase (levels 3 to 4), leucine arylamidase (levels 4 to 5), valine arylamidase (levels 4 to 5), phosphatase acid (levels 3 to 4), naphtol-AS-BI-phosphohydrolase (levels 4 to 5), beta and alpha glucosidase.

Previously, Actinobacteria taxonomy was thought to be related to morphology, which is insufficient to differentiate between different species of many genera.

### 3.4. Identification of the Selected Actinomycete Strains

The four sequences of 16S rRNA gene were analyzed by a comparison with the LEBIBI database [25] and GenBank through Nucleotide BLAST (http://www.ncbi.nlm.nih.gov/BLAST, accessed on 18 June 2021). They belonged to the *Streptomyces* genus, bearing an identity of at least 99% and confirmed our result of a preliminary identification. Nucleotide sequences of partial 16S rRNA of the identified isolates were deposited into Gen-Bank Database (http://www.ncbi.nlm.nih.gov/GenBank, accessed on 18 June 2021) under the accession numbers listed in Table 5.

**Figure 1:**
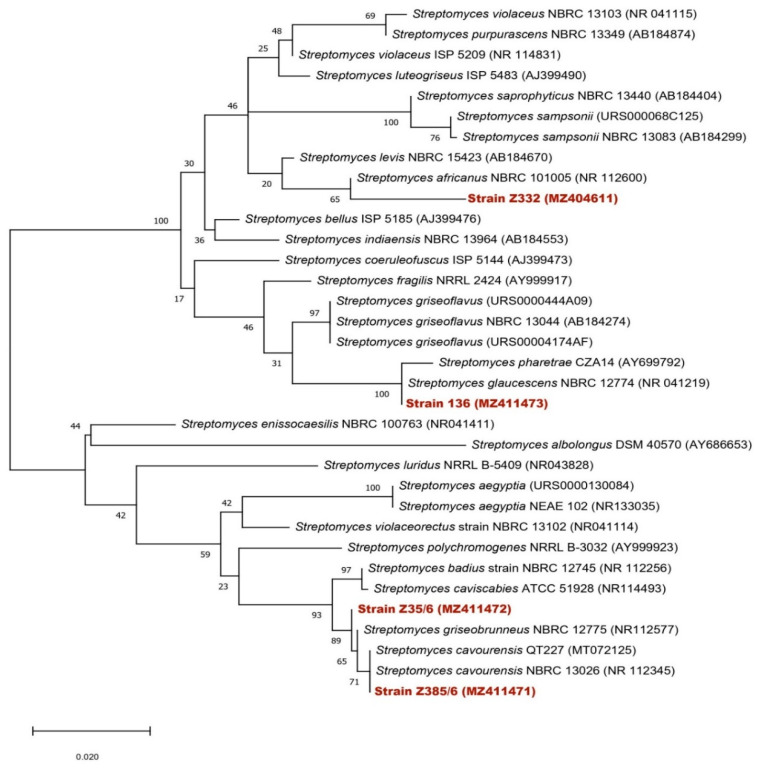
Neighbor-joining phylogenetic tree of the four isolated strains and 30 *Streptomyces* species based on nearly complete 16S rRNA gene sequences (1400 nt). Numbers at nodes indicate levels of bootstrap support (%) based on a neighbor-joining analysis of 1000 resampled datasets. Accession numbers are given in parentheses. Bar, 0.02 nucleotide substitutions per site.

Neighbor-joining trees based on 16S rDNA gene sequences were generated to show the positions of the studied isolates among related isolates (Figure 1), using 1490 nt of aligned sequences and the closest matches to each isolate that were identified on the species level (Table 5). 30 *Streptomyces* species were retrieved from Genbank and used in the construction of the phylogenic tree (Figure 1).

## 4. Discussion

The antibiotic resistance of numerous pathogenic bacteria requires a serious search for new antibacterial agents to fight these pathogens [4]. Secondary metabolites produced by bacteria are still interesting, due to their complicated chemical structures and highly specific antimicrobial activities [5]. The soil bacteria resembling the genus *Streptomyces* are still interesting, being the source of a large number of bioactive natural products and being widely used as antimicrobials [12]; *Streptomyces* species produce about 75% of used antibiotics [31].

In this study, 9.52% of the 210 Actinomycete isolates exhibited efflux pump inhibitor activity. The results show that 6.67% are active against Gram negative bacteria and 2.86% against Gram positive bacteria (Table 1) [9]. Due to their distinctive structure, the thickness of the cell wall and the presence of an outer membrane in Gram negative bacteria explain this sensitivity difference in comparison with Gram negative bacteria [30].

Previously, Actinomycete’s taxonomy was mainly based on morphological and physiological properties, which are insufficient to differentiate between diverse species of many genera [23]. Lately, the identification of the species and phylogenies have usually resulted from 16S rDNA and the use of the polymerase chain reaction (PCR) technique from 16S rDNA sequencing [8]. The comparison of the 16S rDNA sequences of the selected strains Z332, Z35/G, Z385/b and 136 with the GenBank database showed that those isolates belong to the genus *Streptomyces*.

The results of this study, through a screening program of Actinobacteria isolated from specific Moroccan ecosystems, showed the production of bioactive compounds that were natural efflux pump inhibitors and hence of medical interest. This primary screening aimed to highlight the importance of a real refocusing toward the discovery of new natural-bioactive products that may decrease MIC values, improve treatment, minimize toxicity, and reduce the cost of infection treatment. 

## Figures and Tables

**Table 1:** Different MIC and MBC (mg/mL) of the antibiotics used against *E. coli* and *S. aureus*.

	Cloxacilin		Chloramphenicol	
Strains	MIC	MBC	MIC	MBC
SA1199	0.957	0.975	7.81	15.63
SA1199B	62.5	125	7.81	7.81
AG100	250	250	15.63	31
AG100A	250	250	1.95	3.905

MIC: Minimum Inibitory Concentration; MBC: Minimum Bactericidal Concentration.

**Table 2:** The Actinobacteria isolates showing an efflux pumps inhibitory activity against *E. coli* in the absence and presence of the antibiotic.

	**Without Chloramphenicol**		**With Chloramphenicol**	
**Strains**	**AG100**	**AG100A**	**AG100**	**AG100A**
Isolate 4			++	
Isolate 5			++	
Isolate 6			+	
Isolate 7			++	
Isolate 8			+	
Isolate 9			+	
Isolate 10			+	
Isolate 11			+++	
Isolate 12			++	
Isolate 13			+	
Isolate 14			++	
	**Without Cloxacilin**		**With Cloxacilin**	
**Strains**	**SA1199**	**SA1199B**	**SA1199**	**SA1199B**
Isolate 15				+
Isolate 16				+
Isolate 17				++

+: Signs indicate a diameter of the inhibition zone of less than 13 mm, ++: a diameter between 13 and 20 mm, +++: a diameter greater than 20 mm.

**Table 3:** The Actinobacteria strains with an efflux pumps inhibitory activity against *E. coli* and *S. aureus* in the absence and presence of the antibiotic using the confirmatory assay of the agar well diffusion method.

	**Without Chloramphenicol**		**With Chloramphenicol**	
**Strains**	**AG100**	**AG100A**	**AG100**	**AG100A**
Isolate 4			++	
Isolate 5			+	
Isolate 6			+	
Isolate 7			++	
Isolate 8			+	
Isolate 9			+	
Isolate 10			++	
Isolate 11			+++	
Isolate 12			++	
Isolate 13			+	
Isolate 14			++	
	**Without Cloxacilin**		**With Cloxacilin**	
**Strains**	**SA1199**	**SA1199B**	**SA1199**	**SA1199B**
Isolate 15				+
Isolate 16				++
Isolate 17				++

+: Signs indicate a diameter of the inhibition zone of less than 13 mm, ++: a diameter between 13 and 20 mm, +++: a diameter greater than 20 mm.

**Table 4:** Phenotypic characterization of the four selected strains.

Characteristics of *Streptomyces* Strains				
	Z332	Z35/6	Z385/6	136
Spore chain	RF	RF	RF	-
Aerial mass Color				
ISP2	Ochre yellow	beige	ochre brown	ochre yellow
ISP3	light ivory	sepia brown	clay brown	brown beige
ISP4	Ivory	terra brown	ochre brown	honey yellow
ISP5	oyster white	black brown	green brown	traffic yellow
ISP6	Brown beige	green brown	ochre yellow	brown yellow
ISP7	ochre yellow	-	-	maize yellow
Reverse side color				
ISP2	sand yellow	sand yellow	-	
ISP3	Ivory	pale brown	sand yellow	-
ISP4	Colorless	quartz grey	-	brown beige
ISP5	Ivory	-	-	ivory
ISP6	brown beige	khaki grey	-	spares
ISP7	sand yellow	-	-	-
Growth on sole carbon sources				
Glucose	+	++	+	+
Arabinose	+	++	-	+
Sucrose	+	+++	+	+
Xylose	+	++	++	+
Inositol	+	++	+	-
Mannose	+	+	+	+
fructose	+	++	+	+
Rhamnose	+	++	-	-
Raffinose	+	+	-	-
Cellulose	+	+	-	-
NaCl Tolerance	up to 7.5%	up to 7.5%	up to 7.5%	up to 7.5%

+++: High growth; ++: Medium growth; +: Low growth; -: Absence. RF: Rectiflexibiles; ISP: International Streptomyces Project.

**Table 5:** 16S rRNA identification of the four selected isolates.

Strains	16S rRNA Identification	Accession Number
Z385/6	*Streptomyces cavourensis*	MZ411471
136	*Streptomyces glaucescens*	MZ411473
Z332	*Streptomyces africanus*	MZ404611
Z35/6	*Streptomyces griseobrunneus*	MZ411472

## References

[1] Gould K. (2016). Antibiotics: From prehistory to the present day. J. Antimicrob. Chemother..

[2] Shanthi J., Senthil A., Gopikrishnan V., Balagurunathan R. (2015). Characterization of a Potential β -Lactamase Inhibitory Metabolite from a Marine *Streptomyces* sp. PM49 Active Against Multidrug-Resistant Pathogens. Appl. Biochem. Biotechnol..

[3] Breijyeh Z., Jubeh B., Karaman R. (2020). Resistance of Gram-Negative Bacteria to Current Antibacterial Agents and Approaches to Resolve It. Molecules.

[4] Poole K. (2005). Efflux-mediated antimicrobial resistance. J. Antimicrob. Chemother..

[5] Norouzi H., Danesh A., Mohseni M., Khorasgani M.R. (2018). Marine Actinomycetes with Probiotic Potential and Bioactivity against Multidrug-resistant Bacteria. Int. J. Mol. Cell. Med..

[6] Gabashvili E., Kobakhidze S., Chkhikvishvili T., Tabatadze L., Tsiklauri R., Dadiani K., Koulouris S., Kotetishvili M. (2022). Metagenomic and recombinationanalyses of antimicrobial resistance genes from recreational waters of Black Sea coastal areas and other marine environments unveil extensive evidence for their both intrageneric and intergeneric transmission across genetically very diverse microbial communities. Mar. Genom..

[7] Law J.W.-F., Ser H.-L., Duangjai A., Saokaew S., Bukhari S.I., Khan T.M., Mutalib N.-S.A., Chan K.-G., Goh B.-H., Lee L.-H. (2017). Streptomyces colonosanans sp. nov., A Novel Actinobacterium Isolated from Malaysia Mangrove Soil Exhibiting Antioxidative Activity and Cytotoxic Potential against Human Colon Cancer Cell Lines. Front. Microbiol..

[8] Driche E.H., Belghit S., Bijani C., Zitouni A., Sabaou N., Mathieu F., Badji B. (2015). A new *Streptomyces* strain isolated from Saharan soil produces di- (2-ethylhexyl) phthalate, a metabolite active against methicillin-resistant Staphylococcus aureus. Ann. Microbiol..

[9] Nafis A., Elhidar N., Oubaha B., Samri S.E., Niedermeyer T., Ouhdouch Y., Hassani L., Barakate M. (2018). Screening for Non-polyenic Antifungal Produced by Actinobacteria from Moroccan Habitats: Assessment of Antimycin A19 Production by Streptomyces albidoflavus AS25. Int. J. Mol. Cell. Med..

[10] Jakubiec-Krzesniak K., Rajnisz-Mateusiak A., Guspiel A., Ziemska J., Solecka J. (2018). Secondary metabolites of actinomycetes and their antibacterial, antifungal and antiviral properties. Pol. J. Microbiol..

[11] Yunoos M., Sowjanya M., Kumar K.P., Kumar C.A. (2014). Stability indicating. RP-HPLC method for the simultaneous determination of oflox-acin and flavoxate in bulk and pharmaceutical formulations. J. Chem. Pharm. Res..

[12] Fadli M., Chevalier J., Saad A., Mezrioui N., Hassani L., Pages J. (2011). International Journal of Antimicrobial Agents Essential oils from Moroccan plants as potential chemosensitisers restoring antibiotic activity in resistant Gram-negative bacteria. Int. J. Antimicrob. Agents.

[13] Barakate M., Ouhdouch Y., Oufdou K., Beaulieu C. (2002). Characterization of rhizospheric soil Streptomycetes from Moroccan habitats and their antimicrobial activities. World J. Microbiol. Biotechnol..

[14] Samri S., Baz M., Ghalbane I., El Messoussi S., Zitouni A., El Meziane A., Barakate M. (2017). Insecticidal activity of a Moroccan strain of Streptomyces phaeochromogenes LD-37 on larvae, pupae and adults of the Mediterranean fruit fly, *Ceratitis capitata* (Diptera: Tephritidae). Bull. Entomol. Res..

[15] Muñoz-Bellido J.L., Manzanares A.M.A., Andrés M.J.A., Zufiaurre G.M.N., Ortiz G., Hernandez S.M., Garcia-Rodriquez J.A. (1999). Efflux Pump-Mediated Quinolone Resistance in *Staphylococcus aureus* Efflux Pump-Mediated Quinolone Resistance in Staphylococcus aureus Strains Wild Type for gyrA, gyrB, grlA, and norA. Antimicrob. Agents Chemother..

[16] Van Bambeke F., Pagès J., Lee V.J. (2006). Inhibitors of Bacterial Efflux Pumps as Adjuvants in Antibiotic Treat- ments and Diagnostic Tools for Detection of Resistance by Efflux. Recent Pat. Antiinfect. Drug Discov..

[17] Bauer A.W., Kirby M., Sherris J.C., Turck M. (1966). Antibiotic susceptibility testing by a standardized single disk method. Am. J. Clin. Pathol..

[18] Pagès J.-M., Sandrine A.-F., Mahamoud A., Bolla J.-M., Davin-Regli A., Chevalier J., Garnotel E. (2010). Efflux pumps of gram-negative bacteria, a new target for new molecules. Curr. Top. Med. Chem..

[19] Lomovskaya O., Warren M.S., Lee A., Fronko R., Lee M., Blais J., Chamberland S., Renau T., Leger R., Hecker S. (2001). Identification and Characterization of Inhibitors of Multidrug Resistance Efflux Pumps in *Pseudomonas aeruginosa*: Novel Agents for Combination Therapy Identification and Characterization of Inhibitors of Multidrug Resistance Efflux Pumps in *Pseudomonas* a. Antimicrob. Agents Chemother..

[20] Missoun F., de los Ríos A.P., Ortiz-Martínez V., Salar-García M.J., Hernández-Fernández J., Hernández-Fernández F.J. (2020). Discovering low toxicity ionic liquids for Saccharomyces cerevisiae by using the agar well diffusion test. Processes.

[21] Shirling E.B., Gottlied D. (1966). Methods for Characterization of Streptomyces Species. Int. J. Syst. Bacteriol..

[22] Mccarthy A.J., Williams S.T., Grigorova R., Norris J.R. (1990). 17 Methods for Studying the Ecology of Actinomycetes. Methods in Microbiology.

[23] Charousová I., Steinmetz H., Medo J., Javoreková S., Wink J. (2016). Characterization of Antimycins–Producing Streptomycete Strain VY46 Isolated from Slovak Soil. Braz. Arch. Biol. Technol..

[24] Lane D.J. (1991). 16S/23S rRNA sequencing. Nucleic Acids Techniques in Bacterial Systematics.

[25] Flandrois J.P., Perrière G., Gouy M. (2015). leBIBI^QBPP^: A set of databases and a webtool for automatic phylogenetic analysis of prokaryotic sequences. BMC Bioinform..

[26] Saitou N., Nei M. (1987). The Neighbor-joining Method: A New Method for Reconstructing Phylogenetic Trees. Mol. Biol. Evol..

[27] Felsenstein J. (1985). Phylogenies and The Comparative Method. Am. Nat..

[28] Kimura M. (1980). A simple method for estimating evolutionary rates of base substitutions through comparative studies of nucleotide sequences. J. Mol. Evol..

[29] Kumar S., Stecher G., Li M., Knyaz C., Tamura K. (2018). MEGA X: Molecular evolutionary genetics analysis across computing platforms. Mol. Biol. Evol..

[30] Gebreyohannes G., Moges F., Sahile S., Raja N. (2013). Isolation and characterization of potential antibiotic producing actinomycetes from water and sediments of Lake Tana, Ethiopia. Asian Pac. J. Trop. Biomed..

[31] Kumar S., Grefenstette J.J., Galloway D., Albert S.M., Burke D.S. (2013). Policies to reduce influenza in the workplace: Impact assessments using an agent-based model. Am. J. Public Health.

